# CLOCK 3111 T/C SNP Interacts with Emotional Eating Behavior for Weight-Loss in a Mediterranean Population

**DOI:** 10.1371/journal.pone.0099152

**Published:** 2014-06-06

**Authors:** Gemma López-Guimerà, Hassan S. Dashti, Caren E. Smith, David Sánchez-Carracedo, Jose M. Ordovas, Marta Garaulet

**Affiliations:** 1 Department of Clinical and Health Psychology, Universitat Autònoma de Barcelona, Barcelona, Spain; 2 Nutrition and Genomics Laboratory, Jean Mayer US Department of Agriculture Human Nutrition Research Center on Aging, at Tufts University, Boston, Massachusetts, United States of America; 3 Department of Physiology, University of Murcia, Murcia, Spain; 4 Department of Epidemiology, Centro Nacional Investigaciones Cardiovasculares (CNIC), Madrid, Spain; 5 Instituto Madrileño de Estudios Avanzados en Alimentación (IMDEA-FOOD), Madrid, Spain; University of Missouri, United States of America

## Abstract

**Objective:**

The goals of this research was (1) to analyze the role of emotional eating behavior on weight-loss progression during a 30-week weight-loss program in 1,272 individuals from a large Mediterranean population and (2) to test for interaction between *CLOCK* 3111 T/C SNP and emotional eating behavior on the effectiveness of the weight-loss program.

**Design and Methods:**

A total of 1,272 overweight and obese participants (BMI: 31±5 kg/m^2^), aged 20 to 65 years, attending outpatient weight-loss clinics were recruited for this analysis. Emotional eating behavior was assessed by the Emotional Eating Questionnaire (EEQ), a questionnaire validated for overweight and obese Spanish subjects. Anthropometric measures, dietary intake and weight-loss progression were assessed and analyzed throughout the 30-week program. Multivariate analysis and linear regression models were performed to test for gene-environment interaction.

**Results:**

Weight-loss progression during the 30-week program differed significantly according to the degree of emotional eating behavior. Participants classified as ‘very emotional eaters’ experienced more irregular (*P* = 0.007) weight-loss, with a lower rate of weight decline (−0.002 vs. −0.003, *P*<0.05) in comparison with less emotional eaters. The percentage of weight-loss was also significantly higher in ‘non-emotional eaters’ (*P* = 0.009). Additionally, we identified a significant gene-environment interaction associated with weight-loss at the *CLOCK* 3111 T/C locus (*P* = 0.017). By dichotomizing the emotional eating behavior score, linear regression analysis indicated that minor C allele carriers with a high emotional score (> = 11), lost significantly less weight than those C carriers with a low emotional score (<11) (*P* = 0.005).

**Conclusions:**

Emotional eating behavior associates with weight-loss pattern, progression and total weight-loss. Additionally, *CLOCK* 3111 T/C SNP interacts with emotional eating behavior to modulate total weight loss. These results suggest that the assessment of this locus and emotional eating behavior could improve the development of effective, long-tern weight-management interventions.

## Introduction

Lifestyle interventions addressing cognitive behavior and including both dietary and physical activity components are the most effective for the treatment of overweight and obesity [1]–[3]. These interventions achieve short-term weight-loss averaging between 5 to 10 percent, in addition to significant improvements in various health parameters such as blood pressure, cholesterol levels and glycemic control [4]–[6]. However, this modest weight-loss often is followed by subsequent weight regain [2], [7], [8]. As a result, researchers and clinicians now pay close attention to behavioral and psychological factors that may impact the long-term success of weight-loss programs [2], [6], [9]. Factors contributing to low intervention success include emotional eating, previous weight-loss attempts, binge eating, eating disinhibition, body dissatisfaction, lower self-motivation and genetic background [9]–[16].

The association between emotional eating, dietary intake and body weight in particular is becoming an area of increased interest in obesity research [17], [18]. Studies suggest that emotional eaters tend to eat more when experiencing negative emotions such as anger, irritability, fear, sadness, or boredom [19]. As a result, there is a significant association between emotional eating behavior and total weight gain, with a particular increase in the intake of energy dense, nutrient poor foods [9], [20]–[23]. Emotional eaters additionally lose less weight following a weight-loss intervention or program [9]. To complement these results, additional studies are required to assess the role of emotional eating behavior on weight-loss progression and patterns [9], [11], [12], [22].

Abnormalities in the circadian system underlie the development of many illnesses and conditions such as mood disorders, obesity and metabolic syndrome [24]. *CLOCK* (Circadian Locomotor Output Cycles Kaput) is a component of the circadian system, regulating the expression of other integral circadian genes [25], [26]. Studies have found that variants of the *CLOCK* gene are associated with human behaviors and depression, particularly the *CLOCK* 3111 T/C single-nucleotide polymorphism (SNP) [27], [28]. This SNP resides in the 3′ UTR of the gene, and is predicted to affect CTCF transcription factor binding site, as supported by CHIP-seq evidence from the RegulomeDB database [29]. The minor C allele has been associated with psychiatric disorders, shorter sleep duration, increased BMI, higher energy intake and also weight-loss impairment [16], [30]–[32]. Because of the deleterious associations between the minor allele and multiple relevant phenotypes, we hypothesize that *CLOCK* 3111 T/C SNP modulates the association between emotional eating behavior and total weight-loss loss, where minor allele carriers with high emotional scores will experience lower weight-loss than minor allele carriers with low emotional scores.

The first aim of this study was to analyze the role of emotional eating behavior on weight-loss progression, pattern, and total weight-loss during a 30-week weight-loss program in 1,272 individuals from a large Mediterranean population. The second aim was to test for interaction between CLOCK 3111 T/C SNP and emotional eating behavior, on the effectiveness of the weight-loss program as assessed by the total weight-loss at the end of the program.

## Method

### Participants

Between 2008 and 2011, 1,550 subjects voluntarily attended five weight loss clinics in Spain for dietetic and behavioural treatment based upon the principles of a Mediterranean diet [33]. All participants were from the Spanish city of Murcia, located on the Southeast coast of the Mediterranean Sea. Participants were excluded (n = 278) for the following reasons; receiving treatment with thermogenic, lipogenic, or contraceptive drugs; diabetes mellitus, chronic renal failure, hepatic diseases, or cancer diagnosis; bulimia diagnosis, prone to binge eating, or undergoing treatment with anxiolytic or antidepressant drugs; or under the age of 14 or above 75 years. A total of 1,272 overweight and obese subjects (226 men and 1046 women; Mean BMI: 31 SD: 5 kg/m^2^) were finally enrolled in the study. Participants' data were codified to guarantee anonymity. All procedures were in accordance with good clinical practice.

### Ethics

Written informed consent was obtained before subjects were enrolled in the study and was performed in accordance with the Helsinki Declaration of Human Studies and approved by the Ethical Committee of the University of Murcia.

### Intervention

The structure of the program has been described elsewhere in detail [34]. Subjects attended 60-minute therapy sessions once per week. The mean duration of the program was 30-weeks and varied depending on the weight-loss goal. Once the weight-loss goal was achieved, participants followed a five-month maintenance period. Weight was recorded weekly throughout the weight-loss phase of the program. Certified nutritionists led the program sessions. Dietetic treatment was based on the principles of the Mediterranean diet and the distribution of the macronutrient components adhered to the recommendations of the Spanish Society of Community Nutrition [33], [35].

### Measures

#### Emotional eating

Emotional eating was assessed using the Emotional Eating Questionnaire (EEQ), a self-reported questionnaire, administered at the start of the program. The questionnaire was developed and validated directly with Spanish overweight and obese subjects [36]. The questionnaire consists of 10 items designed to assess the extent that emotions affect eating behavior. Examples of the items are: “Do you feel less control over your diet when you are tired after work at night?” and “Do you eat more of your favorite food and with less control when you are alone?” All questions have four possible responses: never, sometimes; generally, and always. Each response was given a score ranging from 0 to 3, with lower scores reflecting healthier behavior.

Initially, subjects were classified into four groups according to scores obtained from the EEQ: (1) scores between 0–5, non-emotional eater; (2) scores between 6–10, low emotional eater; (3) scores between 11–20, emotional eater; and (4) scores between 21–30, very emotional eater. To facilitate additional statistical analyses and future potential clinical application, participants were also dichotomized into emotional and non-emotional eaters using the median emotional score of the population as the cut-off point (<11, non-emotional; ≥11, emotional).

Principal component analysis of the EEQ results identified three factors that explained 60 percent of the total variance in emotional eating. These factors were *Disinhibition* (Factor 1), which includes questions related to loss of control over one's eating behavior; *Type of food* (Factor 2), which includes questions such as, “Is it difficult for you to stop eating sweet things, especially chocolate?” and “Do you crave specific foods?”; and finally *Guilt* (Factor 3), which includes questions such as, “Do you feel guilty when eating ‘forbidden’ foods, such as sweets or snacks?” [36].

#### Anthropometric measurements

Anthropometric measurements were assessed at baseline, and weight was monitored throughout the 30-week program. Participants were weighed while barefoot, wearing light clothes, on a digital scale that measured to the nearest 0.1 kg. Height was measured at baseline using a Harpenden digital stadiometer (rank, 0.7–2.05). Participants were positioned upright and relaxed with the head in the Frankfurt plane. Both height and weight measurements were collected at the same time of the day for all participants. Initial BMI was calculated using baseline measurements as weight (kg) height (m)^−2^. Initial total body fat was measured with bioelectrical impedance, using TANITA TBF-300 (Tanita Corporation of America, Arlington Heights, IL, USA) equipment. Subjects were requested not to drink liquids during the two hours prior to measurement collection. Body fat distribution was also assessed throughout the intervention, and included waist circumference at the level of the umbilicus and hip circumference with the greatest circumference over the greater trochanters. These measurements were used to calculate the waist to hip ratio.

#### Dietary intake

Baseline nutrient intake was determined by means of a 24-hour dietary recall, as described previously [33].

#### Treatment effectiveness

To assess differences in weight-loss progression and pattern over time among the four emotional eating behavior classifications according to the EEQ questionnaire, repeated analysis of covariance (ANCOVA) measurements for weight and week of treatment were used. Data were adjusted for age, sex, and initial BMI with a post hoc Bonferroni adjustment. In order to compare the rate of weight-loss during the program, the slope of the weight-loss curves was calculated for each group.

Moreover, total weight-loss and weight-loss as a percentage of initial weight was calculated to assess the efficacy of the program. Effective treatment classification was based on two parameters: total weight-loss of more than 10 percent of baseline weight, and program attrition as calculated by the percentage of subjects who dropped out of the program prior to reaching a weight-loss of 10 percent of their baseline weight.

#### DNA isolation and CLOCK genotyping

DNA was isolated from blood samples using standard procedures (Qiagen, Valencia, CA, USA). We performed genotyping of the CLOCK 3111T/C SNP using a TaqMan assay with allele-specific probes on the ABI Prism 7900HT Sequence Detection System (Applied Biosystems, Foster City, CA, USA) according to the standardized laboratory protocols.

### Statistical Analyses

Differences in anthropometric measurements and weight-loss between emotional and non-emotional eaters were compared by analysis of covariance (ANCOVA), after adjustment for age, sex, and initial BMI in every approach, except for the association with obesity that was only corrected for age and sex. Correlation coefficients were generated to evaluate relationships between EEQ score and age. A dominant genetic model was applied for the CLOCK 3111 T/C SNP as previously described [32]. Student t test was applied to compare crude means across genotype groups. Multivariate adjustments of the associations by analysis of covariance and estimated adjusted means were performed. All genetic analyses adjusted for sex, age, clinic and study number. All statistical analyses were performed using R statistical software (v. 3.0.0). A two-tailed P-value of <0.05 was considered statistically significant.

## Results

Baseline characteristics of the population studied are shown in Table 1. Briefly, the mean EEQ score in the total sample of 1,272 participants was 11.84 (SD = 5.98). According to the score, about 64% of individuals were classified as ‘emotional eaters’. Additionally, the score was higher in women (M = 12.3, SD = 5.7) than in men (M = 9.19, SD = 6.29) (*P*<0.05), and decreased significantly with age (r = −0.12; *P*<0.0001).

**Table 1:** Initial characteristics of the study population (*n* = 1,272).

Characteristics/Measures	M	SD
Age (yrs)	39.4	12.1
BMI (kg/m^2^)	31.15	5.27
Body fat (%)	37.31	6.62
Waist (cm)	102.2	14.6
Hip (cm)	113.9	10.0
**Dietary Intake**		
Total Energy (kcal/day)	2075	715
Extra calories in snacking (kcal/day)	372	425
Proteins (% total energy)	17.0	4.7
Proteins (g/day)	86.0	33.0
Carbohydrates (% total energy)	41.6	10.5
Carbohydrates (g/day)	214.2	87.8
Fats (% total energy)	42.5	9.4
Fats (g/day)	99.3	44.8
Fiber (g/day)	18.7	11.0
MUFA (% total fat)	55.5	8.0
PUFA (% total fat)	13.7	3.8
SFA (% total fat)	29.9	8.4
**Other Characteristics**	***n***	**%**
BMI≥30 kg/m^2^, n (%)	673	53.6
Drinkers, n (%)	713	56.1
Smokers, n (%)	295	23.2
Sedentary, n (%)	559	44
**CLOCK 3111 T/C SNP**	***n***	**%**
CC, n (%)	104	8.17
CT, n (%)	492	38.7
TT, n (%)	676	53.1

*Note*. Data are presented as mean (M) and standard deviation (SD);

BMI = Body Mass Index; MUFA = Monounsaturated Fatty Acids; PUFA = Polyunsaturated Fatty Acids; SFA = Saturated Fatty Acids.

**Figure 1:**
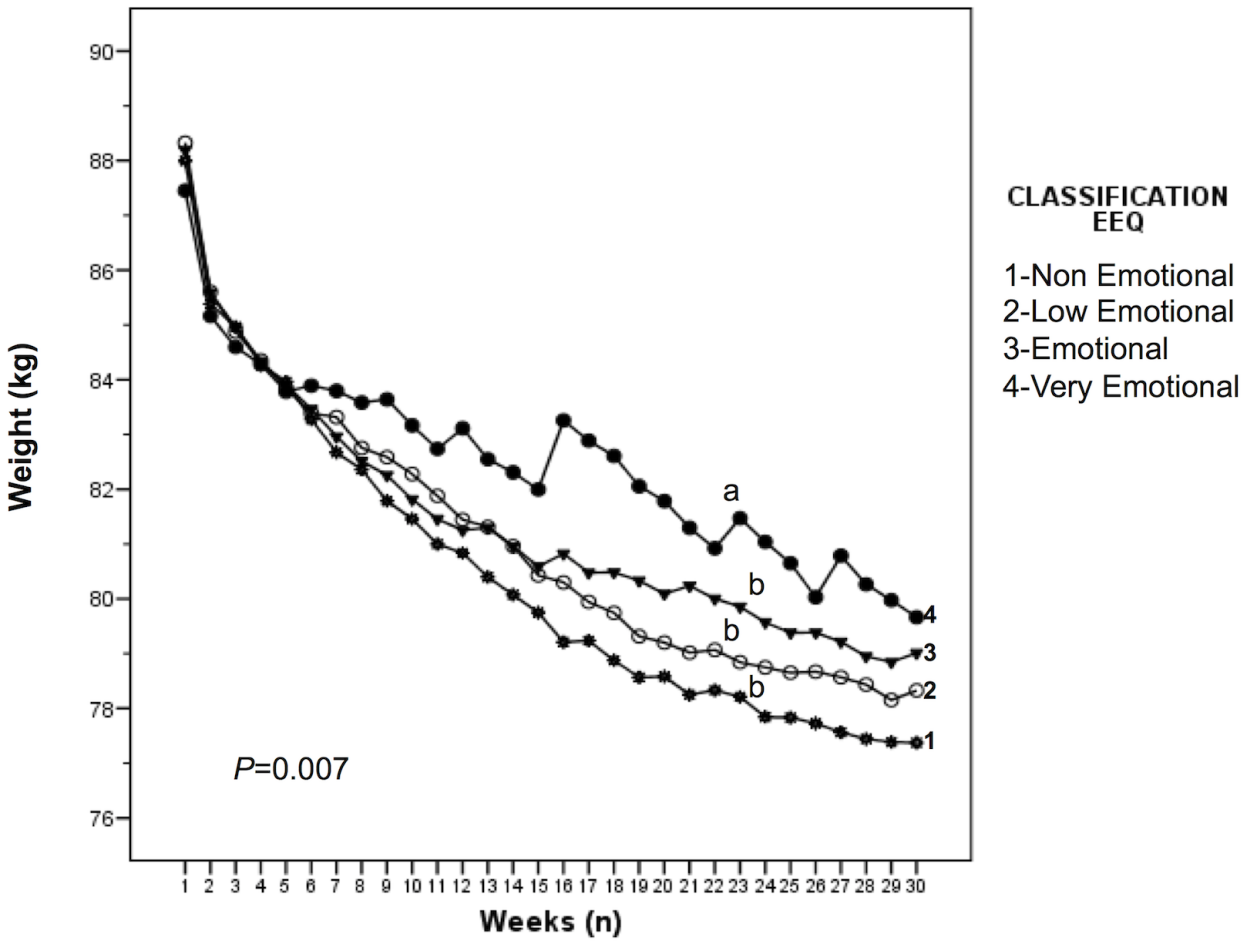
Weight-loss progression during the 30-weeks of treatment stratified by emotional eating based on EEQ classification. Weight-loss analyses within each single time point among the four classifications indicated significant differences exist (*P* = 0.03). Weight-loss progression differed significantly between the ‘very emotional eaters’ group and the three other groups (‘emotional eaters’, ‘low emotional eaters’, and ‘non-emotional eaters’) (*P*<0.05). The rate of weight-loss, as assessed by the slope of the weight-loss curves, also differed between the highly emotional group and the other groups (−0.002 vs. −0.003, *P*<0.05).

Weight-loss patterns differed significantly among the four classification groups (‘non-emotional eaters’, ‘low emotional eaters’, ‘emotional eaters’, and ‘very emotional eaters’) as assessed by a repeated ANCOVA measures adjusted for sex, age, and initial weight for the outcome of weight loss (*P* = 0.007) (Figure 1). Moreover, weight-loss analyses within each weekly time point (ANCOVA) among the four classifications indicated significant differences exist in weight loss (*P* = 0.03). Specifically, *post hoc* Bonferroni analyses indicated that weight-loss progression significantly differed between the ‘very emotional eaters’ group and each of the other three groups (‘emotional eaters’, ‘low emotional eaters’, and ‘non-emotional eaters’) (*P*<0.05) (Figure 1). The rate of weight-loss, as assessed by the slope of the weight-loss curves, also differed between the highly emotional group and the other groups (−0.002 vs. −0.003, *P*<0.05).

Table 2 represents the results from assessing the effectiveness of the program according to the original four levels EEQ classification, as well as the dichotomized classification. In both classifications, treatment was significantly more effective in ‘non-emotional eaters’, where a significantly higher percentage of participants achieved a weight-loss of more than 10 percent of baseline weight. Additionally, EEQ scores were significantly higher among those who dropped out of the program (M = 12.27, SD = 5.91) than those who adhered to treatment and completed the program (M = 11.49, SD = 5.79) (*P* = 0.026).

**Table 2:** Treatment effectiveness in different categories of emotional eating.

	NEE-1	LEE-2	EE-3	VEE-4	P	NEE	EE	P
						n = 385	n = 687	
*Parameters (could be Outcomes)*	*EEQ score classification (Four groups)*					*Clinical classification (Two groups) Or –Classification based on population median (two groups)*		
Subjects achieving weight goal (%)	43.5%	32.3%	28.9%	22.1%	<0.001	36.4%	26.5%	<0.001
Weight loss (% of initial weight)	9.13±0.48^a^	8.17±0.35^a^	8.04±0.28^a^	6.47±0.61^b^	0.009	8.45	7.44	0.007
Attrition (%)	28.8%	35.4%	35.2%	48.5%	0.007	38.9%	41.0%	0.291
Total weight loss (kg)	7.897±0.415^a^	6.980±0.299^a^	6.982±0.241^a^	5.795±0.525^b^	0.021	7.17±0.25	6.54±0.21	0.065

*Note*. EEQ =  Emotional Eating Questionnaire; NEE-1 =  Non-emotional eaters in four groups criterion (EEQ scores between 0–5); LEE-2 =  Low emotional eaters in four groups criterion (EEQ scores between 6–10); EE-3 =  Emotional eaters in four groups criterion (EEQ scores between 11–20); VEE-4 =  Very emotional eaters in four groups criterion (EEQ scores between 21–30); NEE =  Non-emotional eaters median (EEQ scores <11); EE =  Emotional eaters in median criterion (EEQ scores ≥11); *P* = p-values after adjusting for age, sex, and initial BMI. Above – the word clinical implies that you treated them differently (although perhaps this will be your recommendation for the future, that they need different approaches).

When participants were dichotomized into ‘emotional eaters’ and ‘non-emotional eaters’, baseline *measures of obesity and dietary intake* were also different between ‘emotional eaters’ and ‘non-emotional eaters’. ‘Emotional eaters’ had significantly higher BMI (*P* = 0.001), overall energy intake (*P* = 0.023), and carbohydrate intake (*P* = 0.001) than ‘non-emotional eaters’ (Table 3). When we examined the relationship between *CLOCK* 3111 T/C genotype and emotional eating, no significant differences were found in the frequency of the minor C allele between ‘emotional eaters’ and ‘non-emotional eaters’.

**Table 3:** Measures of obesity and dietary intake at baseline in emotional and non-emotional eaters.

Measures	EE (n = 732)	NEE (n = 540)	*P*
BMI (kg/m^2^)	31.46±0.20	30.70±0.23	*0.001*
Body fat (%)	37.6±6.5	36.6±6.9	0.100
Waist (cm)	102±0.5	101±0.6	0.203
WHR	0.89±0.003	0.89±0.003	0.642
Energy intake (kcal/day)	2120.20±32	2006±37	*0.023* 1
Carbohydrates (g)	223±4	204±4	*0.001* 1
Proteins (g)	87±1	83±1	0.106^1^
Fats (g)	100±2	97±2	0.287^1^
Carbohydrates (% of total energy)	42.3	41.0	0.083^1^
Proteins (% of total energy)	16.7	17.2	0.234^1^
Fats (% of total energy)	42.1	42.7	0.362^1^
CC+CT (%)	46.4	47.4	0.917
TT (%)	53.6	52.6	0.923

*Note*. NEE =  Non-emotional eaters in median criterion (EEQ scores <11); EE =  Emotional eaters in median criterion (EEQ scores ≥11); BMI =  Body Mass Index; WHR =  Waist/Hip ratio; *P* =  p-values adjusted for age and sex;

1 p-values adjusted for age, sex, and initial BMI.

**Figure 2:**
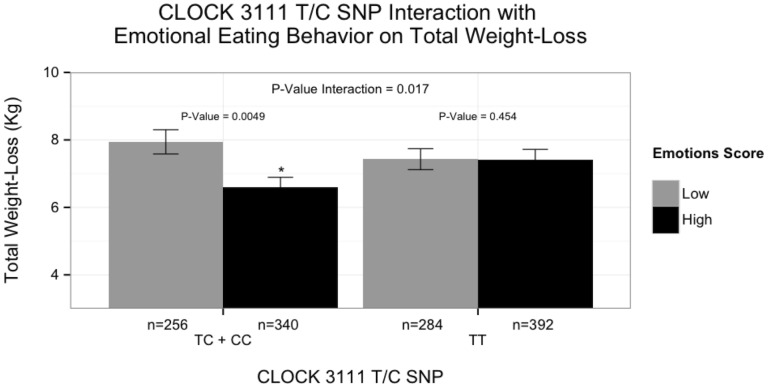
Differences in weight-loss between CLOCK genotypes and emotion eating classification as assessed by EEQ. By dichotomizing the participants into ‘emotional eaters’ and ‘non-emotional eaters’, we found significantly different effects across genotypes at this locus (*P* for interaction  = 0.017). Among minor C allele carriers, ‘emotional eaters’ lost significantly less weight than ‘non-emotional eaters’ (*P* = 0.0049). However, no significant differences were identified between ‘emotional eaters’ and ‘non-emotional eaters’ for total weight-loss among noncarriers (*P* = 0.454). Data are presented as mean ± s.e.m. We used (*) to indicate significant differences between emotional eating groups with the same genotype (*P*<0.050).

Finally, we examined *CLOCK* 3111 T/C genotype in the context of emotional eating and identified a significant gene-environment interaction associated with total weight-loss. By dichotomizing the participants into ‘emotional eaters’ and ‘non-emotional eaters’, we found significantly different effects across genotypes at this locus (β±SE: 1.53±0.64; *P* = 0.017) (Figure 2). Among minor C allele carriers, ‘emotional eaters’ lost significantly less weight than ‘non-emotional eaters’ (β±SE: −1.29±0.46; *P* = 0.0049). However, no significant differences were identified between ‘emotional eaters’ and ‘non-emotional eaters’ for total weight-loss among non-carriers (β±SE: 0.34±0.46; *P* = 0.454). Of note, when we tested for gene-environment interactions between *CLOCK* 3111 T/C genotype and the three different principal component factors of the EEQ (*Disinhibition*, *Type of food*, and *Guilt*) for weight-loss, we found that significance was only achieved for the *Disinhibition* factor in the gene-environment interaction (*P* = 0.002; data not shown).

## Discussion

This prospective, longitudinal study demonstrates that emotional eating behavior influences the effectiveness of weight-loss treatment, by affecting weight-loss progression and patterns. Moreover, the novelty of this result is further enhanced by the original finding suggesting that the 3111 T/C SNP interacts with emotional eating behavior to modulate total weight-loss. Thus, only those ‘emotional eaters’ carrying the minor C allele lose significantly less weight than those classified as ‘non-emotional eaters.’

Studies assessing human circadian systems have identified polymorphisms in circadian clock genes that associate with mood disorders. The *CLOCK* 3111 T/C SNP, located in the 3′-UTR region of the *CLOCK* gene, associates with a higher recurrence rate of major depressive episodes in bipolar depression patients, and with greater insomnia and decreased need for sleep in bipolar patients [27], [37]. Additionally, significant associations between this polymorphism and Attention deficit hyperactivity disorder (ADHD) scores indicate a possible link between *CLOCK* 3111 T/C SNP with adult ADHD [38]. These and other studies suggest that the circadian system, and specifically this genetic variant, have a great impact on mood and behavior.

Furthermore, our previous findings consistently suggest the influence of the circadian system, including this variant, on obesity, obesogenic behaviors, and total weight loss. Studies in several populations indicate that carriers of the minor C allele are more susceptible to higher BMI, have higher energy intakes, higher prevalence of obesity, lower adherence to Mediterranean diet, and experience lower weight-loss [16], [30], [31], [39], [40]. Additionally, variants of core circadian clock genes are associated with greater withdrawal from behavioral weight-reduction program and attrition [41].

Likewise, emotional eating has been previously associated with weight-loss. Individuals classified as low emotional eaters tend to lose significantly more weight than those individuals classified as high emotional eaters [9], [11], [12], [42], [43]. Conversely, emotional eating appears to be associated with more barriers to weight-loss and greater weight-loss difficulties [33], [44]. In accordance with these previous findings, our results indicate that the weight-loss program was significantly more effective in non-emotional eaters, as assessed by total weight-loss and attrition. Furthermore, our results suggest that differences also exist in weight-loss progression and pattern among individuals of different emotional eating behavior classification, as assessed by weekly weight record through the 30-week period (Figure 1). Individuals classified as ‘very emotional eaters’ display the most irregular weight-loss patterns with fluctuating changes in weight, along with lower weight-loss success. This pattern suggests that weight-loss is inconsistent among highly emotional individuals during the 30-week period, possibly resulting in greater withdrawal.

In addition to the longitudinal data from the current study and previous studies that illustrate the importance of emotional eating in weight loss success, the baseline data from the current study are also suggestive. Although no consistent evidence support that obese individuals are more prone to emotional eating than normal weight individuals, our data indicate that at baseline, more than half of the obese individuals (64 percent) are classified as emotional eaters [18], [45], [46]. Moreover, our baseline findings are consistent with previous cross-sectional and experimental studies suggesting that emotional eaters have higher BMI than non-emotional eaters, and consume more sweet and energy-dense foods [19], [21]–[23], [47], [48].

Physiological and psychological mechanisms have been proposed to explain the relationship between emotions and eating behaviors [17], [49]. These theories hypothesize that emotional eaters eat in response to environmental influences instead of hunger cues, and rely on food as a coping strategy for dealing with unpleasant emotions [50]. In response to certain emotions, emotional eaters may be more likely to consume energy dense foods that are high in fat and added sugars, and whose palatability triggers endorphin release that leads to positive shifts in emotion [19], [51]. Although emotional responses may contribute to less healthy eating behaviors, evidence suggests that, the emotion itself is not eliciting dietary intake, instead it is the way emotions are managed that leads to excessive dietary intake [17], [22].

The mechanistic links between genetic variants and eating behaviors may be similarly complex. Various epigenetic and chronobiological mechanisms could explain the interaction between *CLOCK* and emotional eating behavior for weight-loss. Differences in methylation status of CpG sites located in *CLOCK* genes are associated with differences in BMI and weight loss as well as dietary intake [52]. More importantly, these changes are highly related to behavioral changes related to emotions such as constant snacking or eating when bored [52]. Altered methylation status could induce changes in the expression of circadian clock genes, influencing metabolic pathways regulating weight-loss. Alternatively, differences in total weight-loss could be explained by phenotypic differences in chronotypes (eg, behaviors related to morning and evening preferences). Carriers of the CLOCK 3111 T/C minor C allele have reduced sleep duration, elevated ghrelin concentrations and are characterized as evening chronotype [32]. These characteristics, combined with emotional eating behaviors could result in a decreased weight-loss.

Although emotional eating is generally related to less weight loss, closer examination of the components of emotional eating reveals more specific findings. One important barrier to weight-loss is *disinhibition*, an eating behavior associated with reduced ability to control dietary intake, resulting in increased tendency to high caloric intake. Our results indicate that the *CLOCK* 3111 T/C SNP specifically interacts with *disinhibition*, a principal component of emotional eating behavior. This suggests that high *disinhibition* is the main driver for this gene-environment interaction. In fact, previous works have suggested that *disinhibition* is associated with a higher BMI, poor food choices, and low effectiveness of weight-loss programs [53], [54].

The current study had a number of strengths and limitations. This was the first prospective, longitudinal study to assess weight-loss pattern and progression during a 30-week weight-loss program in a large cohort. Additionally, emotional eating was assessed using a questionnaire validated directly with the Spanish overweight and obese population [36]. Study limitations should also be taken into account in interpreting the findings presented here. One limitation is the exclusive use of self-reported data to measure emotional eating. However, the broad definition of ‘emotional eating’ used in the EEQ classification has demonstrated great ability in classifying emotional eaters in the present study. Therefore, the use of EEQ should be confined to healthy subjects with no relevant eating disorders. Findings from the current study have potential implications for future research and clinical practice. Current results imply that assessing EEQ and genetic variation together with other classical approaches such as assessment of energy intake and expenditure, behavioral characteristics related to barriers of weight loss [55] as well as other newer ones such as chronotype assessments [56] or timing of food intake [57] may improve weight-loss.

In conclusion, findings from the current study have possible implications for both future research and clinical practice. Indeed, considering pre-treatment psychosocial assessment that includes eating behaviors, combined with genetic assessment could provide tailored weight-loss recommendations to obese and overweight individuals. These factors may be useful in the development of alternative weight-loss approaches to those at high risk of weight-loss failure [14], [58].
